# The effect of the multimodal intervention including an automatic notification of catheter days on reducing central line-related bloodstream infection: a retrospective, observational, quasi-experimental study

**DOI:** 10.1186/s12879-022-07588-9

**Published:** 2022-07-08

**Authors:** Sohyun Bae, Yoonjung Kim, Hyun-Ha Chang, Sungjin Kim, Hyun-Ji Kim, Hyeyoung Jeon, Juhee Cho, Juyoung Lee, Hwajin Chae, Gyeongmin Han, Shin-Woo Kim

**Affiliations:** 1 Division of Infectious Diseases, Department of Internal Medicine, School of Medicine, Kyungpook National University Hospital, 130 Dongdeok-ro, Jung-gu, Daegu, 41944 Korea; 2 Infection Control Office, Kyungpook National University Hospital, Daegu, Korea

**Keywords:** Infection control, Central venous catheter, Bloodstream infection, Automated medical records system

## Abstract

**Background:**

A central venous catheter (CVC) is an important medical device, but it could be preceding infection and the risk of central line-associated bloodstream infection (CLABSI). CLABSI is a common healthcare-associated infection but results in high cost and mortality; therefore, various efforts to reduce CLABSI have been attempted.

**Methods:**

This is a retrospective, observational, quasi-experimental study in the intensive care unit (ICU) of a single tertiary care hospital. We reviewed and analysed the data of CLABSI rates and days from the insertion to the removal of the temporary CVC between January 2018 and June 2021 with transient periods over 9 months. Sequentially, all patients with the CVC in the ICU underwent the following interventions: maximal barrier precaution, automatic notification of catheter days and 2% chlorhexidine gluconate bathing. A segmented regression analysis of interrupted time series was conducted to compare the CLABSI rates before and after the introduction of multimodal interventions. During study periods, the impact of interventions on CLABSI was evaluated using multivariate logistic regression analyses.

**Results:**

A total of 76,504 patient-days, 28,312 catheter days and 66 CLABSI cases were reviewed in ICU-hospitalised patients. As additional interventions, the CLABSI rate declined from 3.1 per 1000 CVC days to 1.2 per 1000 CVC days in post-interventions. In the pre-intervention and post-intervention periods, 4146 patents had one more short-term CVC. In the multivariate logistic regression analyses, multimodal intervention was one of determinants reducing CLABSI rates (odds ratio (OR), 0.52 [95% confidence interval {CI}, 0.28–0.94]). Indwelling time of CVC over 10 days was the risk factor for CLABSI rates (OR, 6.27 [95% CI, 3.36–12.48]). Of the three interventions, the automatic notification of catheter days was associated with decreased median monthly total CVC days and duration of CVC days per patient.

**Conclusions:**

Multidisciplinary and evidence-based interventions could lead to a decrease in the CLABSI rates. Moreover, the automatic notification of catheter days of the electronic medical healthcare system has shortened the time of indwelling CVC.

**Supplementary Information:**

The online version contains supplementary material available at 10.1186/s12879-022-07588-9.

## Background

Central line-associated bloodstream infection (CLABSI) is a leading serious healthcare-associated infection (HAI) with increasing numbers of immunocompromised patients and invasive procedures and broad antibiotic usage. CLABSI was associated with the increased medical cost, prolonged hospital stay and high mortality of up to 25% [1]. Compared with inpatients without CLABSI, inpatients with CLABSI paid an extra average of 32,000 dollars, and they had a 2.27-fold higher mortality rate [2]. According to the Centers for Disease Control and Prevention (CDC), 25,000 reductions of CLABSIs contributed to saving 414 million excess healthcare costs and 60,000 lives [1]. Fortunately, CLABSIs were preventable HAI with proper aseptic techniques and active surveillance.

Several studies achieved to have zero preventable CLABSI rate after introducing from CLABSI bundle to multifaceted implementations, such as education, surveillance and feedback on results [3–6]. In the meta-analysis involving 2216 adult intensive care units (ICUs), the CLABSI incidence decreased from 5.7 per 1000 catheter days to 2.0 per 1000 catheter days after the implementation of the bundle [6]. The CLABSI bundles were evidence-based and concise interventions to prevent and reduce the CLABSI rates in the ICU [7–9]. Guidelines from the CDC for the prevention of CLABSI emphasised the following five strategies of the CLABSI bundle: hand hygiene; maximal barrier precautions during central venous catheter (CVC) insertion; chlorhexidine antisepsis, excluding the femoral insertion site as possible; and prompt and timely removal of unnecessary CVC [7, 10].

Because the duration of catheterisation is a major extrinsic risk factor associated with the development of CLABSI, efforts to immediately remove a catheter could result in the reduction in the CLABSI rates. Although the prevalence of unnecessary CVC insertion widely ranges from 18 to 39% in a diverse study setting [11], in a hospital-wide survey with 575 admitted patients with one more vascular catheter in a single centre, 21.9% of those had an inappropriate one more vascular catheter [12]. In a multicentre observational study, clinicians described that the unawareness of the presence of a CVC was a major barrier to timely remove it [13]. Therefore, noticing the presence of a CVC to clinicians was likely to be significant to prevent catheter-related infection.

In response, we introduced an automatic notification of catheter days to notify the presence of the CVC and re-evaluate the maintenance of the CVC. This retrospective study aimed to assess the effect of multimodal interventions to the CLABSI rates and analyse the effect of the automatic notification of catheter days. During the study periods, the maximal barrier precautions and 2% chlorhexidine gluconate (CHG) bathing were introduced, and the effect of the multimodal intervention on the CLABSI rates was assessed.

## Methods

### Data collection and study setting

We performed a retrospective, observational, quasi-experimental study in a single tertiary care hospital in Daegu, Republic of Korea. We reviewed and analysed the retrospective data of ICU-hospitalised patients with CVC between January 2018 and June 2021 with a transient period over 9 months during the introduction of multimodal intervention. There were a total of seven adult ICUs with 111 beds, including the medical ICUs, surgical ICUs, stroke units, neurosurgical ICUs, coronary/cardiac care unit, cardiac ICUs and emergency ICUs. We obtained the performance compliance of the CVC checklist, monthly CLABSI cases and monthly CVC days (Additional file 1: Fig. S1). If a patient had two or more CVCs, each CVC was calculated in the CVC days separately. To assess the impact of interventions on CLABSI with considering other covariates, we selected the patients with short-term CVC and obtained data that included the age, sex, comorbidities, ICU type, CVC type, time from admission to insertion of CVC and indwelling time of CVC.

**Fig. 1:**
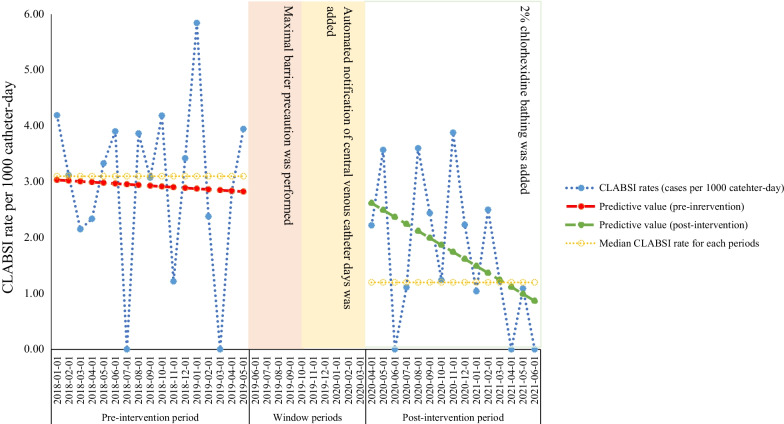
Trend of central line-associated bloodstream infection according to the interventions. The three interventions were performed during the study. The pre-intervention period began in January 2018 and ended in May 2019. Maximal barrier precautions were performed from 1 June 2019. Automated notification of CVC days was performed from 1 October 2019. 2% chlorhexidine bathing was performed from 1 June 2021. Blue line with closed circle indicates monthly CLABSI rates during each period. Yellow line with open circle indicates median monthly CLABSI rates (3.1 CLABSI rates in the pre-intervention period and 1.2 CLABSI rates in the post-intervention period). Red line indicates the predictive value of CLABSI in the pre-intervention period (slope coefficient − 0.01), and green line indicates the predictive value of CLABSI in the post-intervention period (slope coefficient − 0.11). P slope change showed decreasing trend in CLABSI after multimodal interventions, but it was not statistically significant (P slope change = 0.30). CLABSI Central line-associated bloodstream infection

### Intervention

The study was performed in three periods, pre-intervention: from January 2018 to May 2019; transient: from June 2019 to March 2020; and post-intervention: from April 2020 to June 2021. During whole periods, the principal management for reducing CLABSI followed the guidelines for the prevention of intravascular catheter-related infections [7]. The nurse-to-patient ratio was 1:2 to 1:4 in each ICU. Nursing staff and doctors were trained regarding the proper insertion and maintenance of central intravascular catheters. Before CVC insertion or access, healthcare staff washed their hands with soap and rubbed them with alcohol, and then, they performed the procedures aseptically. Two nursing staff belonging to the infection control office monitored the performance compliance of the CVC checklist every 3 months. The CVC checklists consisted of hand hygiene, maximal precaution barrier, antisepsis with disinfectant and CVC catheter site. Doctors inserted a blind CVC without ultrasound guidance.

Doctors performed the procedures with the maximal barrier precautions, including the use of sterile full body drape, mask, cap, sterile gloves and sterile gown on 1st June, 2019. We added the automatic notification of catheter days, which showed the CVC indwelling days in the prescription section of the electronic healthcare system on 1st October, 2019. Medical staff evaluated the need for a CVC every day. Until the assessment of CVC maintenance, the automatic notification of catheter days continued. On 1st April, 2020, all body surfaces were bathed with nonwoven fabric soaked in 2% CHG once daily maintaining the previous interventions.

### Definition

CVCs involved the short- and long-term CVCs. The short-term CVCs were the non-cuffed short-term CVCs, dual-lumen haemodialysis catheters and Swan sheath catheters. The long-term CVCs were the balloon-tipped pulmonary artery central catheters (Swan–Ganz), cuffed and tunnelled catheters (Hickman), implanted central catheters and peripherally inserted central catheters (PICCs).

According to the National Health Safety Network (NHSN) case definition of bloodstream infection (BSI), laboratory-confirmed bloodstream infection (LCBI) was the condition in which patients had a recognised pathogen culture from one or more blood cultures or patients had common skin contaminants from two or more blood cultures on separate sites with clinical symptoms, including fever, chills or hypotension. For these situations, there should not be apparent other sources for bacteraemia [14]. CLABSI is a primary LCBI without another infection in a patient who had CVC at least 48 h before infection [14]. The CLABSI incidence rate was the number of BSI in patients with an indwelling CVC per 1000 CVC days.

### Statistical analysis

Categorical variables are expressed as numbers and percentages. Comparisons of each period were performed for categorical variables using the chi-square test or Fisher’s exact test. Continuous variables are expressed as mean ± standard deviations or median with interquartile ranges (IQRs) and were compared using Student’s t-test or the Mann–Whitney U test. Poisson regression analysis was used to confirm the incidence rate ratio (IRR) and compare the post-intervention CLABSI rate with the pre-intervention CLABSI rate. Segmented regression analyses were performed for estimating the effects of intervention on CLABSI rates in the time series studies. The Kaplan–Meier analysis for CVC early removal was performed using the log-rank test, coding CVC removal within 10 days as a censored event. After excluding patients with the Hickman catheter, implanted port and PICC that typically have long-term indications, we employed multivariate logistic regression analyses to evaluate the risk factor associated with CLABSI in patients with short-term CVCs. A *P* value of < 0.05 was considered statistically significant. All statistical analyses were performed using R statistics ver. 3.1.

### Ethics statement

The Institutional Review Board of Kyungpook National University Hospital reviewed and approved the study protocol (approval numbers: KNUH-202202038). Considering the retrospective nature of the study and the use of anonymous clinical data for analyses, the requirement for informed consent was waived.

## Results

### Trend of the CLABSI rates according to each intervention

Between January 2018 and June 2021, there were 76,504 total patient-days in ICU and 28,312 catheter days in ICU-hospitalised patients. The overall demographic data and CLABSI rates are presented in Table 1. A total of 66 CLABSI cases occurred during the entire study periods (46 in the pre-intervention period, and 20 in the post-intervention period). The median CLABSI rates were 3.1 (IQR, 2.3–3.9) in the pre-intervention period and 1.2 (IQR, 1.1–2.5) per 1000 CVC days in the post-intervention period, which had an IRR of 0.597 (95% confidence interval [CI], 0.359–0.993; *P* = 0.047) in Poisson regression analysis (Table 1). Figure 1 demonstrates the decreasing trends in the CLABSI rates by multimodal interventions (slope coefficient in the pre-intervention period =  − 0.01 vs. slope coefficient in the post-intervention period − 0.11), but the P slope change between the pre-intervention period and post-intervention period was not statistically significant (P slope change = 0.30).

**Table 1:** Overall demographic data and CLABSI rates for each period

Period	CLABSI cases (N)	Total central line days, median (IQR)	Total patient-days, median (IQR)	CLABSI rate^a^, median (IQR)	IRR mean, (95% CI)	*P* value
Pre-intervention period	46	956.0 (856.0–1024.0)	2526.0 (2342.0–2686.0)	3.1 (2.3–3.9)		
Post-intervention period	20	819.0 (802.5–898.5)	2247.0 (2186.0–2375.5)	1.2 (1.1–2.5)	0.597 (0.359–0.993)	0.047

*CLABSI* central line-associated bloodstream infection, *IQR* interquartile range, *CI* confidence interval, *IRR* incidence rate ratio

^a^CLABSI cases per 1000 catheter days

### Study population with indwelling short-term CVCs in the pre-intervention period and post-intervention

Table 2 shows the demographic and clinical data of 4104 ICU-hospitalised patients with short-term CVCs in the pre-intervention and post-intervention periods. Among a total of 4104 ICU-hospitalised patients with short-term CVCs, ICU-hospitalised patients with short-term CVCs were grouped according to CLABSI occurrence. A total of 1593 patients (38.8%) were women, and the median age was 65.0 (53.0–75.0) years. Among 4104 enrolled patients, surgical ICU (71.3%) was the most common ICU, followed by medical ICU and emergency ICU. The jugular vein (48.9%) was the most common insertion site, followed by the subclavian vein (34.0%) and femoral vein (17.1%). In enrolled patients, the median indwelling time of the CVC was 5.0 (3.0–9.0) days.

**Table 2:** Demographic and clinical features of enrolled patients in the pre-intervention period

	Total (N = 4104)	No CLABSI (N = 4048)	CLABSI (N = 56)	P value
Age, median [IQR], y	65.0 [53.0;75.0]	65.0 [53.0;75.0]	64.0 [50.5;71.5]	0.368
Female, n (%)	1593 (38.8%)	1575 (38.9%)	18 (32.1%)	0.371
Time from admission to insertion of CVC, median [IQR], d	1.0 [0.0; 4.0]	1.0 [0.0; 4.0]	0.0 [0.0; 4.0]	0.059
Intensive care unit type, n (%)				
Medical	606 (14.8%)	586 (14.5%)	20 (35.7%)	< 0.001
Surgical	2928 (71.3%)	2893 (71.5%)	35 (62.5%)	0.185
Emergency	570 (13.9%)	569 (14.1%)	1 (1.8%)	0.015
Diabetes mellitus, n (%)	1104 (26.9%)	1088 (26.9%)	16 (28.6%)	0.613
Malignancy, n (%)	717 (17.5%)	706 (17.4%)	11 (19.6%)	0.800
Steroid use during the indwelling time, n (%)	2028 (49.4%)	1998 (49.4%)	30 (53.6%)	0.623
Insertion site				
Jugular	2007 (48.9%)	1996 (49.3%)	11 (19.6%)	< 0.001
Subclavian	1397 (34.0%)	1370 (33.8%)	27 (48.2%)	0.035
Femoral	700 (17.1%)	682 (16.8%)	18 (32.1%)	0.004
Two or more indwelling CVC, n (%)	1383 (33.7%)	1338 (33.1%)	45 (80.4%)	< 0.001
Total indwelling time of CVC, median [IQR], d	5.0 [3.0;9.0]	5.0 [3.0; 9.0]	6.5 [4.0;12.0]	< 0.001
Time from insertion of CVC to occurrence of CLABSI, median [IQR], d	2.0 [0.0;7.0]	–	14.0 [10.0;21.0]	< 0.001
Indwelling time of CVC over 10 days, n (%)	944 (23.0%)	901 (22.3%)	43 (76.8%)	< 0.001
Multimodal intervention	1875 (45.7%)	1860 (45.9%)	15 (26.8%)	0.006

CLABSI, central line-associated bloodstream infection; IQR, interquartile range; CVC, central venous catheter

### Impact of multimodal intervention on CLABSI in the short-term CVCs

As shown in Table 2, there were no significant differences in age, sex, duration from admission to insertion of CVC, diabetes, malignancy and steroid use between the no CLABSI group and CLABSI group. The medical ICU, emergency ICU, subclavian vein insertion site, femoral vein insertion site, two or more indwelling CVC and indwelling time of CVC over 10 days were significantly higher in the CLABSI group than in the no CLABSI group. Jugular vein insertion site and multimodal intervention were significantly lower in the CLABSI group than in the no CLABSI group.

In multivariate logistic analysis, multimodal intervention and jugular vein site were associated with decreased CLABSI (odds ratio [OR] 0.52; 95% CI, 0.28–0.94; *P* = 0.036 and OR 0.37, 95% CI, 0.18–0.71; *P* = 0.005, respectively). Two or more indwelling CVC, and indwelling time of CVC beyond 10 days were associated with increased CLABSI (OR 5.51; 95% CI 2.89–-11.43; *P* < 0.001, and OR 6.27; 95% CI 3.36–12.48; *P* < 0.001, respectively). (Table 3).

**Table 3:** Risk factors for central line-associated bloodstream infection in the intensive care unit

Variable	Univariate logistic regression analysis		Multivariate logistic regression analysis	
	OR (95% CI)	*P* value	OR (95% CI)	*P* value
Medical intensive care unit	3.28 (1.85–5.65)	< 0.001	–	–
Jugular vein site	0.25 (0.12–0.47)	< 0.001	0.37 (0.18–0.71)	0.005
Subclavian vein site	1.82 (1.07–3.09)	0.026	–	–
Femoral vein site	2.34 (1.30–4.06)	0.003	–	–
Two or more indwelling CVC	8.29 (4.43–16.91)	< 0.001	5.51 (2.89–11.43)	< 0.001
Indwelling time of CVC over 10 days	11.55 (6.37–22.45)	< 0.001	6.27 (3.36–12.48)	< 0.001
Multimodal intervention	0.43 (0.23–0.76)	0.005	0.52 (0.28–0.94)	0.036

*OR* odds ratio, *CI* confidence interval, *CVC* central venous catheter

### Effect of the automatic notification of catheter days on CVC days

**Fig. 2:**
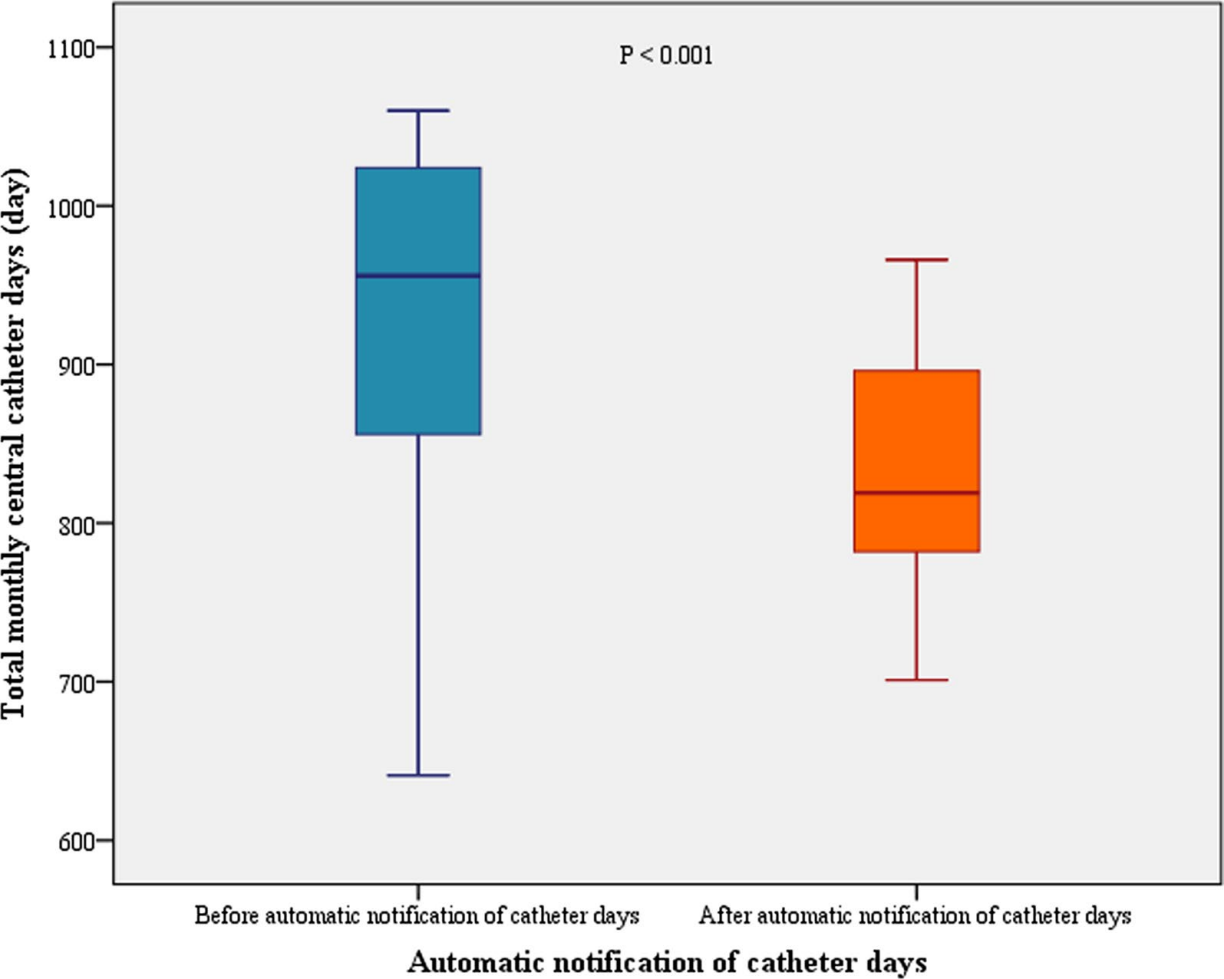
The effect of the automatic notification of catheter days on CVC days. CVC, central venous catheter

**Fig. 3:**
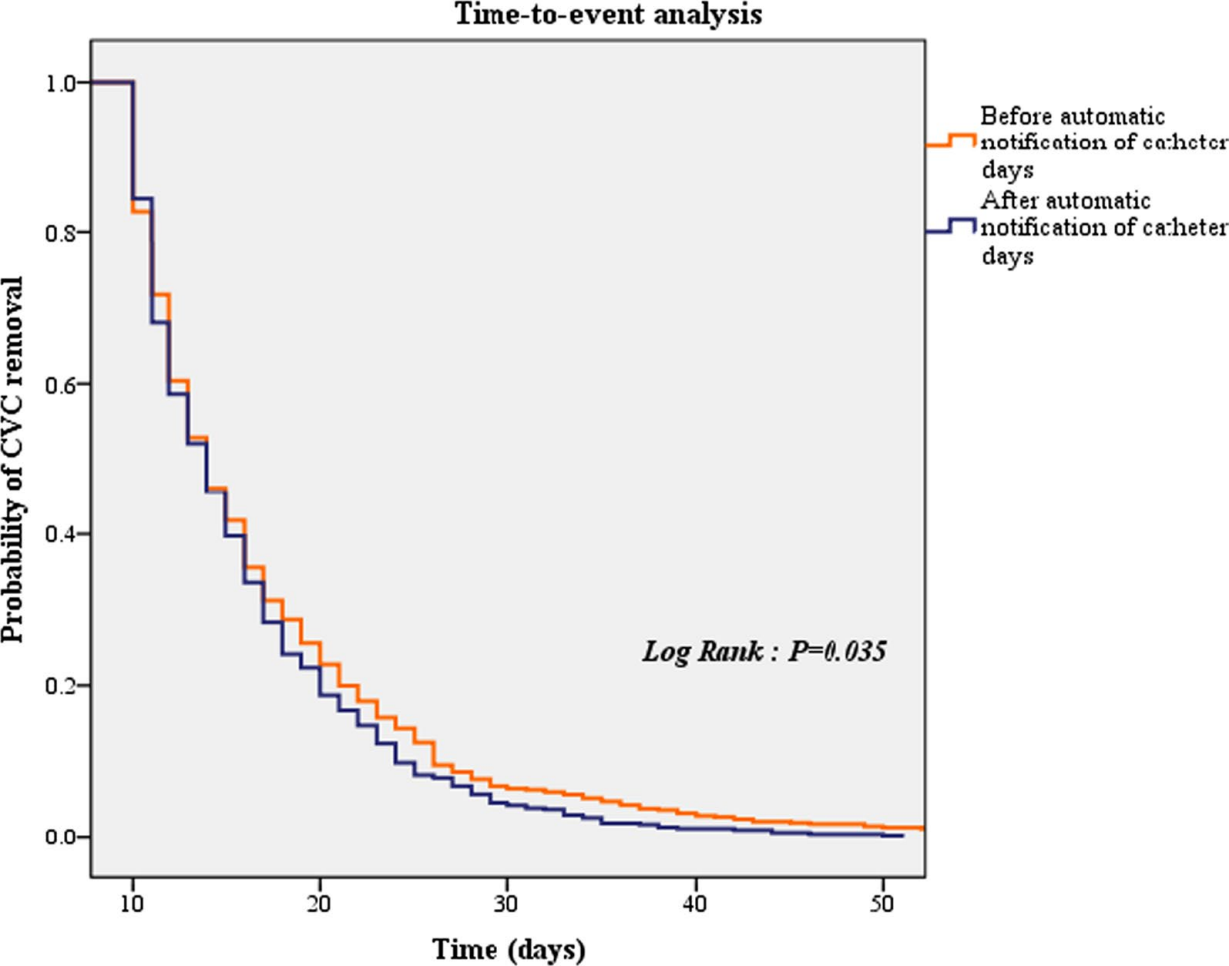
Time-to-event analysis for central venous catheter removal

The median monthly total CVC days were significantly different between the before and after automatic notification of catheter days (median, 956.0 [IQR, 856.0–1024.0] vs. 819.0 [IQR, 782.0–896.0]; *P* < 0.001) (Fig. 2). The duration of short-term central venous catheterisation per patient was significantly different between the before (n = 2680) and after (n = 2595) automatic notification of catheter days (7.53 ± 7.14 vs. 6.74 ± 6.00 days, *P* < 0.001). Time to short-term CVC removal per patient was also decreased after automatic notification of catheter days (Fig. 3) (*P* = 0.035).

## Discussion

This study showed that the total patient-days in ICU (n = 76,504) and catheter days (n = 28,312) and CLABSI rates declined with multifaceted interventions, including maximal barrier precautions, automatic notification of catheter days and 2% CHG bathing. Compared with 3.9 cases per 1000 catheter days in the pre-intervention period, the CLABSI rates decreased by 1.2 cases per 1000 catheter days after the introduction of three interventions, which were lower than 1.83 cases per 1000 catheter days, the fifth percentile of the CLABSI rates through the Korean National Healthcare-associated Infections Surveillance System programme in 2015 [15]. To determine how additional interventions directly affected the CLABSI occurrence would be challenging. However, we introduced the evidence-based tools on the CDC guidelines, which were known to prevent CVC-related infection [7, 16].

In this study, the number of indwelling CVC and indwelling time of short-term CVC beyond 10 days were identified to be risk factors for CLABSI, which is consistent with the findings of the previous study [18–20]. A previous study with 5.6 cases per 1000 catheter days in 896 patients at a single hospital demonstrated that the indwelling time beyond 10 days significantly increased CLABSI cases (OR, 2.867; 95% CI, 1.823–4.507) [18]. Wu et al. showed that the CVC maintenance duration of more than 14 days significantly increased catheter-related bloodstream infection compared with the duration of lower than 14 days (OR, 1.08; 95% CI, 1.04–1.13) [20]. Although Fong et al. suggested that disease severity was related to increased CVC and loss of opportunity for catheter removal [21], 50% of 340 patients had at least one idle CVC day on discharge from ICUs to general wards [22]. In a previous study in non-ICU cases, 89 patients with temporary and non-implanted CVC had idle CVC for a mean of 4.1 days [23]. We continued efforts to reduce the duration of unnecessary central line use with the automatic notification of catheter days and focused on the relationship between the automatic notification of catheter days and duration of indwelling CVC use. This study demonstrated that the introduction of the automatic notification of catheter days in the electronic healthcare system was associated with a reduction in the indwelling time of short-term CVC per patient and reduction in total central line days. This automatic notification of catheter days contributed to decreased short-term CVC indwelling time per patient.

To date, there is no clear and standardised system for unnecessary catheter removal, and the most recommended practices are responsible for combining a CLABSI bundle with multifaceted interventions, including education and feedback [24–26]. Ilan et al. suggested that the removal of non-essential CVCs could be achieved by multidisciplinary team rounding with a checklist including reminders of CVC removal [25]. However, each institution may have different capabilities to team up with committed multidisciplinary experts, including clinicians, infection surveillance nurses and educating clinicians. A multicentre survey-based study in Michigan described that 16.3%–31.1% of 1881 clinicians were unaware of the presence of a CVC, including the medical residents and hospitalists (13.8 vs. 27.3%, respectively) [13]. Because catheter removal is usually at the discretion of the clinicians, the awareness of the presence of a catheter and attending doctor’s decision to remove a catheter may be key point. A previous study in two 26-bed internal medicine clinical teaching units reviewed that the online tool for physician audits of CVC was associated with a significant reduction in catheter days by checking the number of central venous access and reasons for access [27]. Grady found that online tools for CVC management could be an attractive method requiring minimal effort but showing maximal effect [27]. Similarly, the automatic notification of catheter days in our electronic healthcare system simply reminded about the catheter necessity and the catheter indwelling time until the physicians decided on catheter removal. Given that the catheters’ indwelling times were longer in patients outside the ICU than inside the ICU, the daily notification of the presence of a CVC may also elicit the zero CLABSI cases in medical wards [28].

Our study has several limitations. First, we did not consider patient severity and comorbidity, which could be risk factors for CLABSI. Nevertheless, given that PICC would be placed in patients who required venous access for long days, severity and disease could not affect the indwelling time of short-term CVC. Thus, after adjustment for patient’s characteristics, simple sensitisation to clinicians could decrease the duration of catheterisation and CVC infection. Second, we did not collect the prior antibiotic use and number of indwelling CVC for each patient. Moreover, we did not discriminate which catheters caused CLABSI when there were more than two CVCs during CLABSI. However, multiple CVCs increased the risk of catheter-related bloodstream infection in previous studies [29, 30]. And we counted each CVC for the same patient within the catheter days, and our CLABSI rates would be unlikely to be underestimated. Third, because this was a single centre retrospective study, our results were difficult to generalise, and each intervention period had different observation times. Nevertheless, these interventions including the automatic notification of catheter days were principal methods and were easily carried out.

## Conclusion

In summary, the proportion of CLABSI in patients inside an ICU decreased substantially after efforts to reduce contamination, skin coloniser and unnecessary CVCs. Especially, the duration of short-term CVC used decreased after the introduction of the automatic notification of catheter days. To reach the zero CLABSI cases, this study provides the evidence that simple sensitisation of the presence of CVC to physician is attractive for the removal of unnecessary CVCs.

## Supplementary Information

**Additional file 1: Figure S1.** Maximal barrier precautions and 2% chlorhexidinebathing compliance.

## Data Availability

The datasets used during the current study are available from the corresponding author on reasonable request. The data are not publicly available because it contains information that could compromise the privacy of research participants.

## Abbreviations

CLABSI: Central line-associated bloodstream infection
HAI: Healthcare-associated infection
CDC: Centers for Disease Control and Prevention
CHG: Chlorhexidine gluconate
ICU: Intensive care unit
CVC: Central venous catheter
PICC: Peripherally inserted central catheter
LCBI: Laboratory-confirmed bloodstream infection
IQR: Interquartile range
IRR: Incidence rate ratio
OR: Odds ratio
CI: Confidence interval
