# Supplementary material for: The effect of the multimodal intervention including an automatic notification of catheter days on reducing central line-related bloodstream infection: a retrospective, observational, quasi-experimental study
Source: BMC Infect Dis. 2022 Jul 8;22:604. doi: 10.1186/s12879-022-07588-9 (PMC9270824; doi:10.1186/s12879-022-07588-9)
Supplement: Supplementary file 1 — Additional file 1: Figure S1. Maximal barrier precautions and 2% chlorhexidinebathing compliance. [file 12879_2022_7588_MOESM1_ESM.docx]

Additional file 1: Fig S1. Maximal barrier precautions and 2% chlorhexidine bathing compliance
